# Parotid Epithelial–Myoepithelial Carcinoma, Lymph Node Metastasis After 9 Years: A Case Report

**DOI:** 10.3390/reports7040099

**Published:** 2024-11-15

**Authors:** Maria Rita Bianco, Cosimo Galletti, Antonino Maniaci, Giovanni Torrice, Eugenia Allegra

**Affiliations:** 1 Otolaryngology, Department of Health Science, University of Catanzaro, 88100 Catanzaro, Italy; torricegiovanni994@gmail.com (G.T.); eualle@unicz.it (E.A.); 2 Otolaryngology, Department of Medicine and Surgery, University of Enna “Kore”, 94100 Enna, Italy; cosimo.galletti01@unikore.it (C.G.); antonino.maniaci@unikore.it (A.M.)

**Keywords:** epithelial–myoepithelial carcinoma, EMC, salivary glands, epithelial–myoepithelial carcinoma metastasis, epithelial–myoepithelial carcinoma treatment, lymph node metastases

## Abstract

**Background and Clinical Significance**: Epithelial–myoepithelial carcinoma (EMC) is a rare, low-grade malignant tumor of the salivary glands. It is characterized by a low malignancy potential, as indicated by its low rate of lymph node involvement and distant metastasis, and has a local recurrence rate of approximately 50%. Due to the rarity of EMC and the limited data available in the literature, there are no established treatment or follow-up guidelines. **Case Presentation**: We report the case of an 83-year-old man who presented with swelling in the left submandibular region, occurring 9 years following an initial diagnosis of EMC in the ipsilateral parotid gland. After radiological examinations and an ultrasound-guided fine-needle aspiration biopsy, the patient underwent surgical excision of the lesion with a histological diagnosis of recurrence/metastasis of low-grade epithelial–myoepithelial carcinoma. **Conclusions**: This is the first documented case of loco-regional lymph node metastasis 9 years after an initial diagnosis of EMC of the parotid gland. Based on our experience, EMC of the parotid gland, even when diagnosed at an early stage, seems to require a long follow-up period.

## 1. Introduction and Clinical Significance

Epithelial–myoepithelial carcinoma (EMC) is a rare tumor of the salivary glands that primarily develops in the parotid gland and less frequently in the submandibular gland and minor salivary glands [1]. EMC exhibits a low malignant potential, as indicated by its low rates of lymph node involvement and distant metastasis [2]. Generally, EMC of the salivary glands more frequently occurs in older adults, peaking around the seventh decade of life, with a female predominance [3]. The histological structure of EMC typically displays a multinodular growth pattern, mainly consisting of epithelial and myoepithelial cells. The epithelial cells typically form duct-like structures and can exhibit varying degrees of differentiation. The myoepithelial cells surround the epithelial cells, contributing to the tumor’s distinct biphasic histomorphology [2].

EMCs are often described as well-defined masses, white or brown in color, slightly firm or somewhat rubbery, and often encapsulated. They can be asymptomatic or may present with swelling, pain, discomfort, dysphagia, facial nerve weakness, trismus, and hoarseness, with symptoms closely related to the size and location of the tumor [4].

Diagnosis relies on histological examination and is confirmed with immunohistochemical methods: epithelial cells are immunoreactive for low-molecular-weight keratin; and myoepithelial cells are immunoreactive for S-100 protein, muscle-specific actin, and vimentin [5]. Suspicion may arise from imaging studies and procedures such as FNAC (fine-needle aspiration cytology). Contrast-enhanced MRI typically reveals a characteristic multinodular structure with internal septa that allow a differential diagnosis with other salivary gland tumors [6]. FNAC is a valuable but sometimes controversial diagnostic tool, as results can often suggest benign lesions, which may later be identified as EMCs [7,8].

EMCs are generally considered to have a relatively favorable prognosis compared to other malignant salivary gland tumors, but long-term follow-up is essential due to the risk of early-stage recurrence [1].

Due to the rarity of EMC and the limited number of cases reported in the literature, there are currently no established therapeutic guidelines. Complete surgical resection with clear margins is essential for a favorable outcome [7].

We report a case of lymph node metastasis from EMC of the parotid gland occurring more than nine years after the initial diagnosis.

## 2. Case Presentation

An 83-year-old man was referred to our oncological ENT Clinic of the Germaneto Campus of Renato Dulbecco University Hospital in Catanzaro in June 2022 for swelling in the left submandibular region, located posteroinferiorly to the left submandibular gland, which had appeared about six months earlier. Informed consent was requested, and an informed consent form was signed by the patient. He had undergone two ultrasound scans of the neck and salivary glands, two months apart, showing the presence of a highly vascularized neoplasm with internal liquefaction characteristics measuring 0.24 × 0.2 × 0.14 cm, located posteriorly to the left submandibular gland, with an approximate 4 mm increase in size between the two ultrasounds. The patient had chronic HBV infection, hypertension, and atrial fibrillation, had undergone superficial parotidectomy of a left parotid gland neoplasm nine years prior (March 2013) at another facility, and was diagnosed with epithelial–myoepithelial carcinoma, described as follows: “Low-grade epithelial-myoepithelial carcinoma, the multinodular lesion appears encapsulated, margins clear; the associated glandular tissue appears intact”. The patient did not undergo lymph node dissection as the neck was clinically negative, and no adjuvant treatment was performed due to clear resection margins.

**Figure 1:**
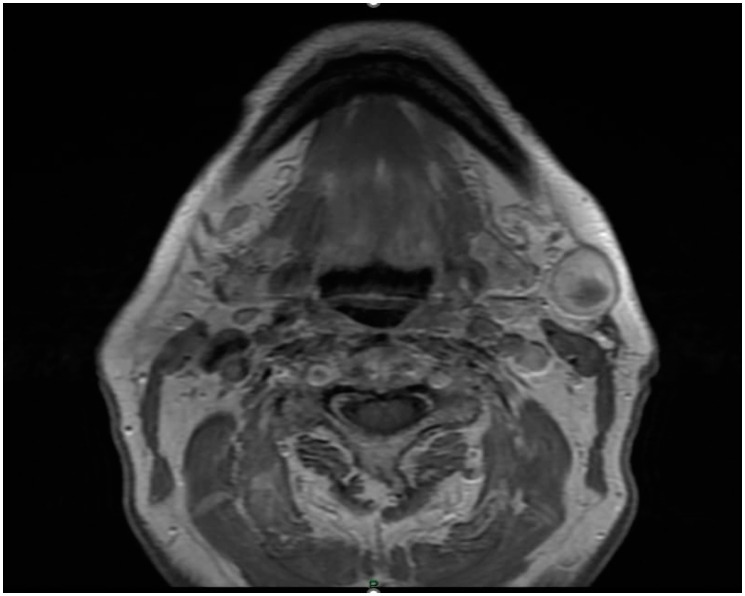
MRI image: a nodulation of approximately 2.3 × 1.6 cm is observed, solid with an internal central area showing fluid signal.

During the gathering of the medical history, the patient reported having undergone follow-up visits for the first five years but then missing subsequent appointments by personal choice. Physical examination revealed a well-circumscribed mass, mobile on both superficial and deep planes, not spontaneously painful or tender to palpation. For better characterization of the mass, an ultrasound-guided fine-needle aspiration biopsy was performed, which was reported as non-diagnostic. An MRI with contrast was then performed, which revealed “posteroinferiorly to the left submandibular gland, the presence of a nodulation approximately 2.3 × 1.6 cm, solid with a central internal area showing fluid signal with signal restriction in Diffusion Weighted Imaging (DWI) and heterogeneous enhancement with contrast media” [Figure 1]. No suspicious lymph nodes indicative of disease involvement were observed on this MRI scan. Additionally, a chest CT scan was performed, and no lesions suspicious for metastases were identified. Given these characteristics, the decision was made to proceed with surgical removal of the lesion without neck dissection, as there was no evidence of malignancy of the lesion. This approach was also deemed appropriate considering the patient’s comorbidities and the inability to discontinue anticoagulant therapy.

**Figure 2:**
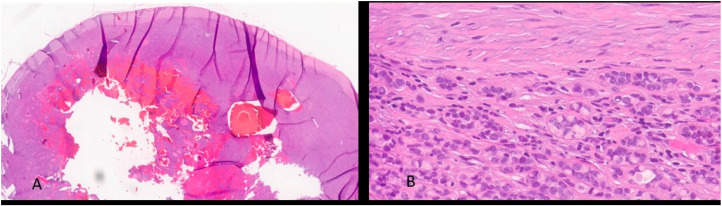
H&E-stained section. (**A**) (2 HPF magnification) shows an expansive, invasive nodular neoplasia characterized by bland central necrosis and hemorrhagic areas, surrounded by a hypercellular peripheral zone. (**B**) (40 HPF magnification) shows some histological and cytological details of neoplasia composed of myoepithelial epithelioid, plasmacytoid or spindle cell, single or aggregate in nests, or solid and trabecular structures.

**Figure 3:**
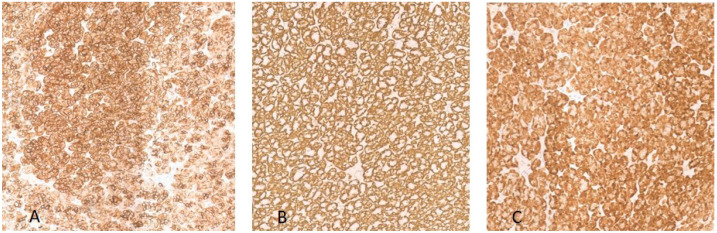
Immunohistochemical neoplasia panel of antibodies (10 HPF). (**A**) shows intense membrane cytokeratin 7 staining of the neoplastic cell. (**B**) shows cytoplasmatic smooth muscle actin staining of the neoplastic cell. (**C**) shows diffuse cytoplasmatic and nuclear S100 staining of the neoplastic cell.

The post-operative histological examination showed the following: “a whitish nodular lesion of 2.5 × 2 × 2 cm, centrally hemorrhagic. Microscopy revealed a neoplastic tubular proliferation with scant reticular stroma with lymphoid elements in the context. Presence of neoplastic tubules with slight nuclear atypia and a continuous basal layer, with expression of cytokeratin 7, pancytokeratin, AML, S100, and CitoA. Additional morphological findings included the presence of a thick continuous capsule and PAS+ mucous secretion. DIAGNOSIS: morphological findings consistent with metastasis of low-grade epithelial-myoepithelial carcinoma” [Figure 2 and Figure 3]. The patient was referred to a tumor board but was not considered suitable for adjuvant treatment due to advanced age (83 years) and comorbidities.

The patient is currently enrolled in our oncological follow-up protocol, including monthly check-ups for the first six months, with quarterly neck ultrasounds and MRI with contrast every six months. From the second to the fifth year, check-ups will be quarterly, with semiannual ultrasounds and annual MRIs. From the third year onwards and for the next ten years, follow-ups will be semiannual, with semiannual neck ultrasounds and annual MRIs. Currently, the patient shows no signs of recurrence or metastasis two years after the surgical intervention.

## 3. Discussion

Epithelial–myoepithelial carcinoma is a tumor with a high probability of loco-regional recurrence. Diagnosis typically relies on histopathology and immunohistochemistry since fine-needle aspiration often fails to provide a definitive diagnosis.

The primary treatment for EMC of the salivary glands involves radical surgical resection followed by adjuvant radiotherapy (RT), particularly when surgical margins are narrow or lymph node metastases are detected [9]. However, the role of adjuvant radiotherapy remains unclear, as the literature reports conflicting data [2,10], and there is no consensus on its true impact on the prognosis of this tumor.

The literature reports a local recurrence rate for EMC ranging from 11.8% to 50% and a lymph node metastasis rate of 15% to 20% [11,12,13].

In a study of 61 patients with EMC, Seethala et al. [14] reported a local recurrence rate of 36.3% and a distant metastasis rate of 5.2%. Kasper et al. [15] noted a significant local recurrence rate ranging from 31.3% to 43%, with most recurrences occurring within 5 years. The most common sites of distant metastasis are the lungs and bones [16].

Noel et al. [17] documented a case of pulmonary metastasis of epithelial–myoepithelial carcinoma of the salivary glands 14 years after the excision of the primary tumor and 11.5 years after the second and final recurrence.

In a 2021 study, Nakaguro et al. [18] reported that despite 30–50% of individuals experiencing local recurrence, lymph node and distant metastases are rarely observed.

In 2007, Seethala et al. [14] found a five-year survival rate of 93.5% in their study of 61 patients with EMC.

In 2018, Gore [19] examined the survival of patients with epithelial–myoepithelial carcinoma across all EMC sites, identifying 5-, 10-, and 20-year survival rates of 72.7%, 59.5%, and 38.3%, respectively. The rates of lymph node metastasis (4.2%) and distant metastasis (2.6%) were low. Survival was significantly reduced in individuals with T2, T3, and T4 or M1 tumors compared to those with T1 or M0 tumors.

In 2015, Vazquez et al. [2], examined a sample of 246 patients with EMC and found disease-specific survival rates at 60, 120, and 180 months to be 91.3%, 90.2%, and 80.7%, respectively. Distant metastases occurred in 4.5% of cases. Patients with low-grade histology had significantly better survival rates compared to those with high-grade tumors. Additionally, patients with lesions larger than 4 cm had the poorest survival outcomes.

Although EMC is described as a low-grade malignancy, fatal cases have been reported in the literature [15,16,17].

Overall, EMC demonstrates a variable but generally favorable survival rate, which depends on the tumor’s characteristics. Achieving complete surgical excision with clear margins is crucial for a positive prognosis. Adjuvant radiotherapy can be beneficial in cases with positive margins for achieving loco-regional control [7,8,9,10,11,12,13,14,15,16,17,18,19,20]. Despite the low risk of lymph node metastasis reported in the literature, our case is the first to document loco-regional lymph node metastasis occurring 9 years after the initial diagnosis of EMC of the parotid gland.

## 4. Conclusions

EMC of the parotid gland, even when diagnosed at an early stage, seems to require a long follow-up period.

## Data Availability

The original contributions presented in this study are included in the article. Further inquiries can be directed to the corresponding author.
